# Using the center of pressure movement analysis in evaluating spontaneous movements in infants: a comparative study with general movements assessment

**DOI:** 10.1186/s13052-023-01568-8

**Published:** 2023-12-20

**Authors:** Halil Ibrahim Celik, Ayse Yildiz, Ramazan Yildiz, Akmer Mutlu, Ruhi Soylu, Kivilcim Gucuyener, Aysu Duyan-Camurdan, Esin Koc, Eray Esra Onal, Bulent Elbasan

**Affiliations:** 1 Bilge Çocuk Special Education and Rehabilitation Center, Beysukent, Çankaya, s06800 Ankara Turkey; 2 Faculty of Health Sciences, Department of Physiotherapy and Rehabilitation, Erzurum Technical University, Erzurum, Turkey; 3 Faculty of Physical Therapy and Rehabilitation, Developmental and Early Physiotherapy Unit, Hacettepe University, Ankara, Turkey; 4 Faculty of Medicine, Department of Biophysics, Hacettepe University, Ankara, Turkey; 5 Faculty of Medicine, Department of Pediatrics, Section of Pediatric Neurology, Gazi University, Ankara, Turkey; 6 Faculty of Medicine, Department of Pediatrics, Section of Social Pediatrics, Gazi University, Ankara, Turkey; 7 Faculty of Medicine, Department of Pediatrics, Section of Neonatal Medicine, Gazi University, Ankara, Turkey; 8 Faculty of Health Sciences, Department of Physiotherapy and Rehabilitation, Gazi University, Ankara, Turkey

**Keywords:** Center of pressure, General movements assessment, Spontaneous movements, Quantitative movement analysis, Cerebral palsy

## Abstract

**Background:**

Researchers have attempted to automate the spontaneous movement assessment and have sought quantitative and objective methods over the past decade. The purpose of the study was to present a quantitative assessment method of spontaneous movement using center-of-pressure (COP) movement analysis.

**Methods:**

A total of 101 infants were included in the study. The infants were placed in the supine position on the force plate with the cranial-caudal orientation. In this position, the recording of video and COP movement data were made simultaneously for 3 min. Video recordings were used to observe global and detailed general movement assessment (GMA), and COP time series data were used to obtain quantitative movement parameters.

**Results:**

According to the global GMA, 13 infants displayed absent fidgety movements (FMs) and 88 infants displayed normal FMs. The binary logistic regression model indicated significant association between global GMA and COP movement parameters (chi-square = 20.817, *p* < 0.001). The sensitivity, specificity, and overall accuracy of this model were 85% (95% CI: 55–98), 83% (95% CI: 73–90), and 83% (95% CI: 74–90), respectively. The multiple linear regression model showed a significant association between detailed GMA (motor optimality score-revised/MOS-R) and COP movement parameters (F = 10.349, *p* < 0.001). The MOS-R total score was predicted with a standard error of approximately 1.8 points (6%).

**Conclusions:**

The present study demonstrated the possible avenues for using COP movement analysis to objectively detect the absent FMs and MOS-R total score in clinical settings. Although the method presented in this study requires further validation, it may complement observational GMA and be clinically useful for infant screening purposes, particularly in clinical settings where access to expertise in observational GMA is not available.

**Supplementary Information:**

The online version contains supplementary material available at 10.1186/s13052-023-01568-8.

## Background

Spontaneous movements are simple or complex involuntary movement patterns produced by the young nervous system without sensory input and exist from early fetal life until the end of the first half-year postterm [1]. These movements, which are an integral part of the fetal and infantile typical development, have important functions such as constantly changing the fetal position, providing sensory stimulations, and supporting skeletal, muscular, and sensorimotor neural development. In the early postnatal period, they are crucial for the development of gross motor skills and exploratory sensorimotor behavior that enable an infant to explore its body and environment [2, 3].

General movements (GMs), a part of the spontaneous movement repertoire, are complex and variable entire-body movements involving variable sequences of the neck, trunk, leg, and arm movements [4]. Their richness and complexity provide information about the functional integrity of the young nervous system. The crucial role of GMs in early brain development and its long-term relevance for the later development of cognitive, speech-language, and motor functions has been increasingly recognized, especially in high-risk infants. Therefore, the expert-based observation of GMs, known as Prechtl’s General Movement Assessment (GMA), is usually used for the developmental evaluation and early detection of neurodevelopmental disabilities [5–9]. The GMA, when performed during the fidgety movements (FMs) period, is the most sensitive and specific indicator for later disabilities, particularly cerebral palsy (CP) [7, 10].

The GMA is a qualitative method that analyzes video recordings of spontaneous movements based on gestalt perception of normal vs. abnormal movement patterns [4, 11]. Although it is a valid and reliable assessment tool, GMA is vulnerable to human factors (e.g., having a qualitative structure, requiring well-trained and experienced observers, and being sensitive to observers’ fatigue) like all human-powered assessments [12–15]. Therefore, also with the effect of technological advancement, researchers have attempted to automate the GMA and have sought quantitative and objective methods over the past decade [16, 17]. These methods using an automated approach can be categorized into 3D motion capture [18–20], computer-based video analysis [15, 21–23], accelerometer [14, 24, 25], and electromagnetic motion tracking [26, 27]. Yet, recent research has emphasized that while automated approaches have the potential to expand the clinical application of the GMA, they may only be useful to complement, but not to replace human assessment in clinical practices [16, 28]. In a systematic review, it was found that automated approaches to predicting motor impairment in infants have a pooled sensitivity and specificity of 73% and 70%, respectively, and it was stated that these approaches remain inferior to observational GMA [28]. In another systematic review, it was noted that although automated approaches were feasible, their validation was limited [16].

Given the advantages and disadvantages of previous automated methods, in the present study, we studied an automated method using the center of pressure (COP), which is the projection of the center of mass (COM) on the support surface and is generally used in the indirect evaluation of COM movement. The analysis of COP movement, which can be used in the evaluation of spontaneous movements in infants, reflects not only COM movements but also the kinematics and dynamics of movements of body parts [29, 30]. The present study expanded on these considerations and aimed to present a quantitative method that performs global and detailed GMA with COP movement data. We hypothesized that the analysis of COP data during spontaneous movements in the supine position would be useful in detecting an age-specific spontaneous movement pattern, that is FMs, for clinically feasible GMA prediction in infants.

## Methods

This study was designed as cross-sectional. Ethical approval for the study was obtained from the Gazi University Ethical Committee (GO 2019–210). Informed consent was obtained from all families of the infants included in the study.

### Participants

A convenience clinical sample of preterm and term infants (for pooling both the normal and abnormal FMs patterns) was prospectively included. Term infants who were born at a gestational age of ≥ 37 weeks were recruited from among those with standard follow-up in the Gazi University Faculty of Medicine Hospital Pediatric Clinics. Preterm infants who were born at a gestational age of < 37 weeks were recruited from among those with standard follow-up in the Gazi University Faculty of Medicine Hospital Newborn Medicine Clinics. The infants included in the study (1) had the post-term age between 9 and 20 weeks; (2) without any genetic/metabolic syndrome and orthopedic deficit; and (3) used no medications, such as anticonvulsants, that can affect movements. A total of 101 infants were included in the study (See: supplementary file 1). Subsequently, the infants were assigned to Normal FMs and Absent FMs groups according to the global GMA results. In the final analyses, the normal FMs group consisted of 88 infants (40 girls and 48 boys) with a mean post-term age of 14.1 weeks (± 2.9), and the Absent FMs group consisted of 13 infants (8 girls and 5 boys) with a mean post-term age of 12.6 weeks (± 3.2).

### Data collection procedures

After the sociodemographic and birth data were obtained from the hospital database, the infants were placed in the supine position on the force plate (Kistler Type 9260AA, Kistler Instruments AG, Winterthur, Switzerland) covered with foam padding for the infants’ comfort, with the cranial-caudal orientation of the infant aligned parallel to the length of the force plate. In this position, the recording of video and COP time series data of spontaneous movements were made simultaneously for 3 min. Video recordings were obtained using a digital video camera (Panasonic Lumix DMC-TZ20, Osaka, Japan) on a tripod placed in front of the infants (See: supplementary file 2)*.*

Spontaneous movements were recorded in the awake, active, and non-crying states, in accordance with the GMA methodology [31] The recordings were made at least 2 h after feeding, in a quiet, illuminated, and warm room, and, while the infant was naked or partly dressed, if possible. The infant did not see his/her parents and there was no visual or auditory stimulus (such as a mirror or a toy) around that attracted attention. If the infant cried, fussed, or rolled during data collection while lying supine, the recordings were interrupted and, if possible, repeated at another time convenient for the infant. Video recordings were used for global and detailed GMA, and COP time series data were used to obtain quantitative movement parameters of the COP.

### General movements assessment

While Prechtl’s method [4] was used for the global GMA, the Motor Optimality Score-Revised (MOS-R) was used for the detailed GMA [31]. Prechtl’s method is a valid and reliable qualitative video observation method in which video recordings of spontaneous movements are analyzed based on gestalt perception and provide information about the quality of GMs and the functional integrity of the young nervous system [4]. At 3–5 months of post-term age (FMs period), Prechtl’s method focuses on whether FMs are present or not. FMs are continuous, tiny, and elegant movements, characterized by small amplitudes, moderate speed, and variable accelerations of neck, trunk, and limbs in all directions. The Prechtl’s method classifies the GMs patterns including normal FMs, absent FMs, and abnormal FMs [7]. The MOS-R, also known as detailed GMA, is a semi-quantitative assessment that evaluates the concurrent repertoire of movement and postural patterns as well as FMs and gives a total score [32]. The MOS-R total score is the sum of scores in the following five sub-categories: (i) temporal organization and quality of fidgety movements, (ii) observed movement patterns other than fidgety movements, (iii) age-adequate movement repertoire, (iv) observed postural pattern, and (v) movement character. The maximum total score indicating the best performance is 28, while the minimum is 5 [31].

The video recordings taken during the FMs period were evaluated by two experienced pediatric physiotherapists (B.E and A.M.) who were certified in basic and advanced GMA and were blinded to the participants' clinical histories and neurological conditions. Global and detailed GMA were performed independently in separate runs of the same video recordings. In case of disagreement in the Global GMA (four infants' recordings; 3.96%), a consensus was reached based on additional evaluations. When the disagreement in the detailed GMA involved only one point, the higher score was accepted. If the disagreement involved more than one point, the video recordings were re-evaluated until a consensus was reached on a final score. Previous studies have reported high intra-class correlation coefficients (ICCs) of inter-observer reliability (ranging between 0.80 and 0.98) [33, 34]. The ICC of inter-observer reliability for the total MOS-R was 0.988 (95% CI = 0.982–0.992) in the present study.

### COP data analysis

Although many measuring devices are used for COP, which is the projection of the COM on the support surface and is used to indirectly evaluate the COM movement, force platforms are considered the gold standard [35]. In the present study, a Kistler force plate interfaced with a computer system running MARS® data acquisition software was used for the analysis of COP movement. The 3-min COP time series (raw) data of infants exhibiting spontaneous movement while lying on their backs were recorded on the computer. Considering the fact that the spontaneous movement frequency in infants was below 3 Hz [36], the analog signals obtained from the force platform were digitized using a sampling frequency of 50 Hz in order to stay a factor of 10 above the highest frequency of the movement. The COP time series data were processed using a customized MATLAB software program (Version 2018b / Natick, Massachusetts, USA) for filtering and calculation of COP movement parameters. A 'Fourth Order 10 Hz Low-Pass Butterworth' filter was applied for filtering any noise likely to be recorded by the force plate [37]. In the calculation of COP movement parameters, the first 10 s were taken as the time for the infants to acclimate to the ground of the force platform, so the raw signal of the last 170 s was used. All COP movement parameters were calculated from the COP time series in the medial–lateral (X) and caudo-cephalic (Y) directions, and the resultant (R). This process outputs the COP position of the infant with respect to real-world coordinates relative to the location of the four sensors and the bounds of the force plate. COP movement parameters and their mathematical formulas, each of which captures different characteristics of the spontaneous movements, [38, 39] are given in supplementary file 3.

### Statistical analysis

Because of the differences in the current height and weight between the normal and absent FMs groups, COP movement parameters were normalized for each infant’s current height and weight. The normal distribution analysis of the data was examined using visual (histogram and probability graphs) and analytical methods (KS-SW tests). The association between global GMA results (dichotomous variable) and COP movement parameters (numeric variables) was examined with the point-biserial correlation test, and the relationship between numerical variables was examined with the Spearman or Pearson correlation test, as appropriate. Binary logistic regression analysis was performed to predict the global GMA results (normal FMs or absent FMs), and multiple linear regression analysis was performed to predict the detailed GMA results (numeric variables) using the possible COP movement parameters determined in previous analyses. COP movement parameters, which were significantly correlated with the global and detailed GMA results with a coefficient of above 0.25, were examined in terms of multicollinearity, and only one clinically significant parameter showing multicollinearity (correlation coefficient > 0.70) was included in the model. The predictive performance metrics of the logistic regression model, such as sensitivity and specificity, were obtained from the classification table. For the multiple linear regression analysis, the non-normally distributed variables were transformed to approximate normal distribution using logarithmic transformation. All statistical analyses were conducted with IBM SPSS Statistics 26.0 (SPSS Inc., Chicago, IL, USA) with the alpha equal to 0.05 [40].

## Results

According to the global GMA, the infants were allocated into two groups: the normal FMs group (*n* = 88) and the absent FMS group (*n* = 13). The clinical and demographic characteristics of the infants are presented in Table 1. There were significant differences between groups in terms of preterm birth, birth weight, gestational age, and current weight and height. While the infants in the normal FMs group did not have any perinatal pathology, those in the absent FMs group had grade III intraventricular hemorrhage (*n* = 4, 31%), grade II hypoxic/ischemic encephalopathy (*n* = 2, 15%), chronic lung disease (*n* = 2, 15%), hyperbilirubinemia (*n* = 6, 46%), necrotizing enterocolitis (*n* = 3, 23%), and neonatal sepsis (*n* = 2, 15%).

**Table 1:** Demographic and clinical characteristics

	**Total** **(*n* = 101)**	**Normal FMs (*n* = 88)**	**Absent FMs (*n* = 13)**	**p**
Female n (%)	48 (47.5)	40 (45.5)	8 (61.5)	0.278^α^
Preterm birth*,* n (%)	14 (13.9)	8 (9.1)	6 (46.2)	**0.002**^**α**^
Gestational age (wks)*,* median (25th/75th centiles)	38.4 (37.6–39.3)	38.6 (37.8–39.3)	37.6 (35.1–39.1)	**0.048**^**β**^
Post-term age (wks)*,* median (25th/75th centiles)	14 (11.6–16.3)	14.3 (11.7–16.4)	11.6 (10.6–14.4)	0.063^β^
Birthweight (g)*,* median (25th/75th centiles)	3150 (2905–3455)	3175 (2950–3490)	2550 (1990–3150)	**0.003**^**β**^
Current weight (g)*,* median (25th/75th centiles)	6340 (5750–6850)	6390 (5775–7085)	5850 (5150–6770)	**0,045**^**β**^
Current height (cm)*,* median (25th/75th centiles)	62 (60–65)	63 (61–65)	60 (57–62)	**0.003**^**β**^

p^α^: Pearson's *chi*-*squared* test, p^β^: Mann–Whitney U test

### COP movement parameters

When the COP movement parameters of the groups were compared, there was a significant difference in instantaneous velocity R (Std), approximate entropy X, Y, and R (*p* = 0.035, 0.044, 0.001, and 0.002, respectively). COP movement parameters exhibiting a significant association with the global GMA were instantaneous velocity R (Std) (r = -0.213, *p* = 0.032), instantaneous velocity R (Skewness) (r = 0.216, *p* = 0.030), approximate entropy X, Y, and R (*r* = 0.304, 0.405, and 0.397; *p* = 0.002, < 0.001, and < 0.001, respectively). COP movement parameters with a significant association to the detailed GMA were instantaneous velocity R (Std), (RMS), and (Skewness), instantaneous velocity Y (Std) and (RMS), velocity range Y and R, total distance Y and R, instantaneous distance RMS Y and R, ellipse metrics, average distance, and approximate entropies (See: supplementary file 4).

### Prediction of the global GMA

The binary logistic regression model performed in two steps with the forward stepwise method was generally significant and at least one of the independent variables in the model was a significant predictor (chi-square = 20.817, *p* < 0.001). In addition, it was found that the goodness of fit of the model (Hosmer–Lemeshow test; *p* = 0.255) was sufficient. It was determined that instantaneous velocity R (Std), which measures velocity variability, and approximation entropy R, which measures complexity, were significant variables in predicting the global GMA (normal FMs or absent FMs) and they explained 35% of the cumulative variance in the dependent variable (global GMA) (Nagelkerke R^2^ = 0.347). A 1-unit decrease in instantaneous velocity R (Std) increased the risk of absent FMs by 1%, while a 1-unit increase in approximate entropy R increased this risk by 2.44 times (Table 2). The sensitivity, specificity, and overall accuracy of the model were 85% (95% CI: 55–98), 83% (95% CI: 73–90), and 83% (95% CI: 74–90), respectively. Furthermore, the positive and negative predictive values were 42% (95% CI: 30–55) and 97% (95% CI: 91–99), respectively.

**Table 2:** Prediction of Global GMA with COP movement parameters using logistic regression

Parameters	n	OR	%95 CI	B	Standard error	p
**Instantaneous velocity R (Std)**	101	0.990	0.981–0.999	-0.010	0.005	**0.032**
**Approximate entropy R**	101	2.447	1.376–4.353	0.895	0.294	**0.002**
**Constant**	-	0.035	-	-3.354	1.336	**0.012**
${\text{P}}({\text{Y}})=\frac{1}{1+{{\text{e}}}^{-(-3.354+-0.01*\text{Instantaneous Velocity R}\left({\text{std}}\right)+0.0895*\text{Aproximate Entropy R})}}$. Classification threshold = 0.15						

*OR* odds ratio, *CI* confidence interval, *B* unstandardized coefficient, *P(Y)* probability of the absent FMs

### Prediction of the detailed GMA

It was determined that at least one of the independent variables in the multiple linear regression model, which was performed in two steps with the backward stepwise method, was a significant predictor and the model was generally significant (F = 10.349 and *p* < 0.001). In predicting the MOS-R total score, instantaneous velocity R (Std), instantaneous velocity R (Skewness), and approximate entropy R variables were significant predictors and they explained 24% of the cumulative variance in the dependent variable (R^2^ = 0.242). A 1-unit logarithmic increase in instantaneous Velocity R (Std) and a 1-unit logarithmic decrease in instantaneous Velocity R (Skewness) and approximate entropy R created a 1-unit logarithmic increase of 0.17, 0.22, and 0.27 points in the MOS-R, respectively (Table 3). The MOS-R total score was predicted with a standard error of approximately 1.8 points (6%).

**Table 3:** Prediction of total MOS-R with COP movement parameters using linear regression

Parameters	n	B	95% CI	p
**Instantaneous velocity R (Std)**	101	0.172	0.045/0.299	** < 0.001**
**Instantaneous velocity R (Skewness)**	101	-0.219	-0.404/-0.035	**0.009**
**Approximate entropy R**	101	-0.265	-0.406/-0.125	**0.020**
**Constant**	-	1.294	0.933/1.655	** < 0.001**
Standard error of the prediction = 1.82 ± 1.02 The dependent variable: log (MOS-R) The regression equation = 1.294 + 0.172* log (Instantaneous Velocity R (Std))-0.219*log (Instantaneous Velocity R (skewness))-0.265*log (Approximate Entropy R)				

*CI* confidence interval, *B* unstandardized coefficient

## Discussion

In the current study, we studied the feasibility and potential of COP movement analysis in the evaluation of infant spontaneous movements. The quantitative method presented in this study was found to classify absent FMs and normal FMs at a clinically acceptable level with an overall accuracy of approximately 83% and to predict the MOS-R total score with an error of approximately 1.8 points (6%).

### COP movement analysis

Previous studies obtaining COP data with a pressure-sensitive mat have reported that COP movement analysis in supine position was feasible and that there were different COP movement patterns between preterm and term infants [30, 41, 42]. However, the researcher provided no evidence of the association of COP data with GMA, which is widely used for developmental assessment and early detection of later disabilities. In a study using a pressure-sensitive mat, Kulvicius et al. stated that the machine learning method that handles pressure data obtained in the supine position classified absent and normal FMs movement patterns with 81% accuracy [43]. However, some disadvantages of pressure-sensitive mats are noted. In obtaining COP data, force plates, not pressure-sensitive mats, are accepted as the gold standard measurement tool [35]. Moreover, since infants usually flex their extremities during spontaneous movements, the movements of the extremities in the air cannot be directly captured by the pressure-sensitive mats. All of these findings implied that COP movement analysis in supine position is appropriate for infants, yet COP data should be obtained by a force plate, similar to the method used in our study.

### Global GMA

Over the last decade, the clinical and scientific value of GMA has been increasingly recognized and it has been revealed to be the most sensitive and specific predictor of later disabilities [7, 10]. The current international guidelines emphasize that GMA during the FMs period has the best predictive value and accuracy for the early detection of CP and high-risk CP [44, 45]. In a systematic review, it was reported that the method with the best predictive power for early CP diagnosis was Prechtl’s GMA (sensitivity: 97%, specificity: 89%) performed in the FMs period [10]. Furthermore, as its associations with later cognitive, speech-language, and motor functions in addition to CP have become more evident, the merits of GMA for early detection of disabilities have been increasingly recognized [5–8].

Given the GMA during the FMS period is a strong predictor for neurodevelopmental disorders, it is extremely important to automate GMA and make it easily accessible. Therefore, with the help of today's technological advancement, researchers have sought automated methods that are easy to use, widely utilized in clinics, independent of the user/observer, and do not interfere with infant movements [16, 17], and they have suggested several methods. Adde et al. used the general movements toolbox (GMT), a computer-based video analysis method, to distinguish between absent FMs and normal FMs classified according to the observational GMA, and noted that absent FMs were predicted with sensitivity and specificity values of 82% and 70%, respectively [15]. In another study of the same research group, it was reported that GMT predicted absent FMs with lower sensitivity (80%) and specificity (53%) compared to the previous study [46]. Machireddy et al. performed a 3D spontaneous movement analysis using a hybrid system that includes an advanced wearable sensor (3D-accelerometer, 3D-gyroscope, and 3D-magnetometer) and computer-based video analysis methods. They stated that absent FMs and normal FMs were classified with an accuracy of 84% using a machine-learning algorithm [47]. Gao et al. evaluated the spontaneous movements of 1–6 month-old infants with an accelerometer, regardless of FMs and writhing periods, and found that abnormal and normal GMs were discriminated with an accuracy of 80% [14]. In the previous study, the inclusion of abnormal GMs belonging to two different GMA periods, such as poor repertoire GMs and absent FMs with quite different CP predictive values, in the same group made the interpretation of the results difficult. On the other hand, some limitations of the wearable sensors and GMT used in previous studies raise concerns for clinical use. As previously mentioned, in observational GMA, the infant is in the supine position and untouched, moving free of any external stimulus, and should also be in an appropriate behavioral state. However, it is not known whether these sensors affect infants' spontaneous movements or the infant's wearing procedures, during which the infant has to be touched or manipulated, and often time-consuming could affect the infant's behavioral state in wearable sensor-based approaches [17]. In addition, although the definition of GMs includes the movements of all body parts, sensor-based approaches usually consider the movements of the arms, legs, and head, not the trunk movements, which indicates that they provide incomplete information about the full-body movement. GMT requires special setup and evaluation conditions, and it is difficult to detect small and fast changes in movements due to 2D video recordings [23, 48, 49]. In the present study, an automated method using the COP was presented and values of predictive power comparable to previous studies were obtained in the classification of normal FMs and absent FMs (accuracy: 83%, sensitivity: 85%, specificity: 83%). The increase and decrease in the instantaneous velocity R (Std) (velocity variability) were interpreted in favor of normal FMs and absent FMs, respectively. This result supports the reduced variability in absent FMs [4, 11]. In addition, the increase in the approximate entropy R, which evaluates the movement complexity, increased the risk of absent FMs. It can be inferred that infants exhibiting absent FMs, unlike the complexity in normal FMs, have a chaotic or excessive movement complexity. Moreover, we claim that this presented method has some advantages. First, it is non-intrusive, which means no sensor attachment is necessary. Second, in contrast to method requring sensory attachment, it deals with the global movement of body parts, not separately, in accordance with the linguistic definition of GMs. Lastly, it is easy to use and does not requiring a special setup and lab environment.

### Detailed GMA

To our knowledge, though there are studies in the literature on the prediction of the detailed GMA (MOS-R) during the FMs period with a quantitative method, there exist studies examining the association of the detailed GMA with the early diagnosis of CP and minor neurological dysfunction (MND) and motor, cognitive, language-speech, and behavioral performance [13–18].

It was stated that MOS total and sub-category scores are associated with gross and fine motor development at 12 months, and the evaluation of motor repertoire in addition to global GMA was useful in detecting abnormal motor development [34, 50]. Kwong et al. found that MOS-R was associated with CP diagnosis and neurodevelopment at 2 years [51]. Yang et al. investigated the association between motor repertoire and functional mobility level in children with CP and reported that the higher the MOS total score, the better the functional level [52]. Einspieler et al. emphasized that motor repertoire evaluation provides information about the clinical phenotype of CP [31]. Hitzert et al. reported that MOS was associated with cognitive and behavioral performance at the age of 6 years in healthy-term children [53]. Bruggink et al. found that children with MND aged 7–11 years had higher total MOS scores than the CP group and lower than those of the typically developing group [54]. Örtqvist et al. investigated the association between motor repertoire and neurological outcomes at 12 years of age and reported that MOS-R increased the sensitivity and specificity of global GMA [55]. Considering the association of motor repertoire with short- and long-term neurodevelopmental status, the MOS-R has the potential to become part of the toolbox of early infant assessment, and consequently, the decision-making process of early intervention. However, it is noteworthy that there is no study in the literature on the prediction of the MOS-R with a quantitative method. The advantages of quantitative methods, such as ease to use, high reproducibility, and user-/observer-independency, have created the need to predict the MOS-R with a more objective and automated assessment method. The quantitative method presented in this study was found to predict the MOS total score with an error of approximately 1.8 points, which is approximately 6% of the maximum score (28 points). Instantaneous velocity R (Std), instantaneous velocity R (Skewness), and approximate entropy R showed that velocity variability, smooth/fluent movement, and complexity contribute to the prediction of the MOS total score, respectively. All these movement features are compatible with the observational evaluation of the MOS-R [31]. However, we should also note that the MOS-R includes items that do not reflect in head or body movements including mouth movements, tongue movements, and smiles; therefore, COP may not fully reflect these small and rapid movements.

### Limitations

The present study had some limitations. First, the study sample did not include infants with abnormal FMs, another pattern of the FMs period. Second, observational GMA results were predicted in this study. Longitudinal studies may examine the prediction of the definitive diagnosis of CP at 2–3 years of age using the quantitative method presented in this study. Thirdly, further psychometric studies are needed to evaluate the applicability of this method in populations with different risk factors to make it generalizable and adaptable, though the initial results are promising. Lastly, adding new measurement parameters such as periodicity to the evaluation of the COP movement and the use of machine learning algorithms in the prediction of the observational GMA may contribute to the prediction power of this method.

## Conclusions

The quantitative method presented in this study is relatively inexpensive, non-intrusive (i.e. no marker on the infant’s body), easy to use, and user-/observer-independent. It addresses the global movement of body parts, not separately, in accordance with the linguistic definition of GMs. This method classified absent FMs and normal FMs at a clinically acceptable level with an overall accuracy of approximately 83%. In addition, the MOS-R total score was predicted with a low error rate of 6%. These findings suggest possible avenues for using COP movement analysis to objectively detect absent FMs and MOS-R total score in clinical settings. The method presented in this study is not an alternative to observational GMA and requires further validation, yet may be clinically useful for infant screening purposes, particularly in clinical settings where access to expertise in observational GMA is not available. In addition, it may provide a way to determine infants with absent FMs and refer them to age-specific individual early intervention as early as possible.

### Supplementary Information

**Additional file 1.** Flow diagram of study participants. Shows the number of participants who were evaluated, excluded, and did not complete the study.

**Additional file 2.** The evaluation setting: placement of the force platform and camera. Shows the clinical setting in which the assessments were conducted.

**Additional file 3.** COP movement parameters and their mathematical formulas. Shows Calculations for the COP movement parameters.

**Additional file 4.** Group comparisons for COP movement parameters. Shows group comparisons of COP movement parameters in Normal FMs and Absent FMs groups.

## Data Availability

The datasets used and/or analysed during the current study are available from the corresponding author on reasonable request.

## Abbreviations

COP: Center-of-pressure
GMA: General movement assessment
FMs: Fidgety movements
MOS-R: Motor optimality score-revised
GMs: General movements
CP: Cerebral palsy
COM: Center of mass
GMT: General movements toolbox
MND: Minor neurological dysfunction
