# Supplementary material for: Using the center of pressure movement analysis in evaluating spontaneous movements in infants: a comparative study with general movements assessment
Source: Ital J Pediatr. 2023 Dec 20;49:165. doi: 10.1186/s13052-023-01568-8 (PMC10731817; doi:10.1186/s13052-023-01568-8)
Supplement: Supplementary file 1 — Additional file 1. Flow diagram of study participants. Shows the number of participants who were evaluated, excluded, and did not complete the study. [file 13052_2023_1568_MOESM1_ESM.docx]

## Assessed for eligibility

Had the chronological age between 9 and 20 weeks;

Without any genetic/metabolic syndrome and orthopedic deficit;

Used no medications, such as anticonvulsants, that can affect movements.

## (n=126)

## Excluded (n=15)

## Not meeting inclusion criteria

## bbbbbbbbbb

**n=111**

## Unable to complete assessments (n=10)

-Sleepiness, crying, prolonged hiccups (n=4)

-Rolled to one side (n=6)

**Whose data was analyzed**

**(n=101)**

**Supplementary Figure 1:** Flow diagram of study participants
