# Supplementary figures and images for: Using the center of pressure movement analysis in evaluating spontaneous movements in infants: a comparative study with general movements assessment
Source: Ital J Pediatr. 2023 Dec 20;49:165. doi: 10.1186/s13052-023-01568-8 (PMC10731817; doi:10.1186/s13052-023-01568-8)

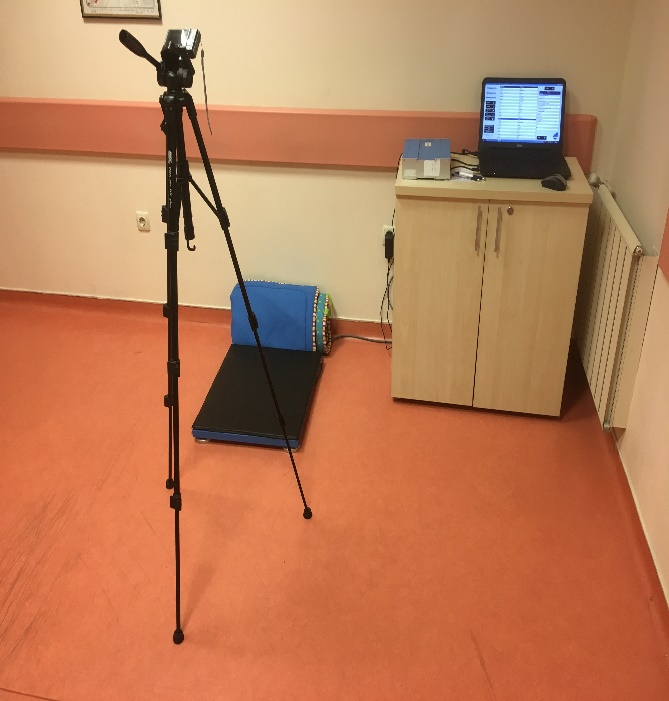

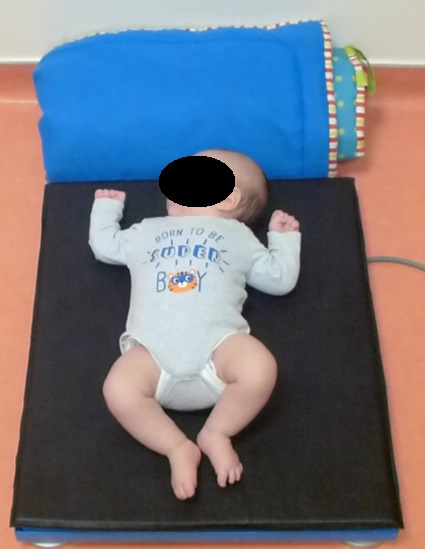


**Supplementary Figure 2:** The evaluation setting: placement of the force platform and camera

Supplement: Supplementary file 2 — Additional file 2. The evaluation setting: placement of the force platform and camera. Shows the clinical setting in which the assessments were conducted. [file 13052_2023_1568_MOESM2_ESM.docx]
