# Supplementary material for: Using the center of pressure movement analysis in evaluating spontaneous movements in infants: a comparative study with general movements assessment
Source: Ital J Pediatr. 2023 Dec 20;49:165. doi: 10.1186/s13052-023-01568-8 (PMC10731817; doi:10.1186/s13052-023-01568-8)
Supplement: Supplementary file 3 — Additional file 3. COP movement parameters and their mathematical formulas. Shows Calculations for the COP movement parameters. [file 13052_2023_1568_MOESM3_ESM.docx]

| **Parameters’ name** | **Units** | **Equation** | **Explanation** |
| --- | --- | --- | --- |
| **Instantaneous velocity Std** | mm/s | $\sqrt{\frac{1}{N-1}\sum_{1}^{N} {{(V}_{(k+1)}- V_{mean})}^{2}}$ | Variability of velocity |
| **Instantaneous velocity RMS** |  | $\frac{1}{N}\sqrt{\sum_{1}^{N} {{(V}_{(k+1)}- V_{mean})}^{2}}$ | Variability of velocity |
| **Instantaneous velocity Skewness** |  | $\frac{E{(V-V_{mean})}^{3}}{\sigma^{3}}$ | Outlier of velocity |
| **Average velocity** | mm/s | Total distance / Total time | Distance traveled in one second |
| **Instantaneous acceleration Std** |  | $\sqrt{\frac{1}{N-1}\sum_{1}^{N} {{(A}_{(k+1)}- A_{mean})}^{2}}$ | Variability of acceleration |
| **Instantaneous acceleration RMS** |  | $\frac{1}{N}\sqrt{\sum_{1}^{N} {{(A}_{(k+1)}- A_{mean})}^{2}}$ | Variability of acceleration |
| **Instantaneous acceleration Skewness** |  | $\frac{E{(A-A_{mean})}^{3}}{\sigma^{3}}$ | Outlier of acceleration |
| **Total distance X** | mm | $\sum_{k=1}^{N-1} {(X}_{(k+1)}- X_{k})$ | Total distance traveled on the X-axis |
| **Total distance Y** | mm | $\sum_{k=1}^{N-1} {(Y}_{(k+1)}- Y_{k})$ | Total distance traveled on the Y-axis |
| **Total distance R** | mm | $\sum_{k=1}^{N-1} \sqrt{{(X_{(k+1)}- X_{k})}^{2}+{(Y_{(k+1)}- Y_{k})}^{2}}$ | Total distance traveled on the R-axis |
| **Instantaneous distance RMS** |  | $\frac{1}{N}\sqrt{\sum_{1}^{N} {{(X}_{(k+1)}- X_{mean})}^{2}}$ | Variability of instant distance traveled |
| **Ellips area (95% CI)** | mm^2^ | Area = $\pi\times a \times b$  a = $\sqrt{3\times(\mathrm{StdX}^{2}+ \mathrm{StdY}^{2}+D)}$  b = $\sqrt{3\times(\mathrm{StdX}^{2}+ \mathrm{StdY}^{2}-D)}$  D=$\sqrt{{(StdX}^{2}+\mathrm{StdY}^{2})-4(\mathrm{StdX}^{2}x\mathrm{StdY}^{2}-\mathrm{StdXY}^{2})}$ | The area of the ellipse that includes 95% of the COP positions. |
| **Range** |  | \|$Max - Min\vert$ | Difference of largest and smallest values |
| **Approximate Entropy** |  | $ApproxEnt=\Phi m-\Phi m+1$  $\Phi m={(N-m+1)}^{-1}\sum_{i=1}^{N-m+1} log(Ni)$ | Complexity |
| **Velocity** | mm/s | $V=\frac{\Delta X}{T}$ | Position change per unit time |
| **Acceleration** | mm/s^2^ | $A=\frac{\Delta V}{T}$ | Velocity change per unit time |
| Std: Standart deviyasyon (σ), RMS: Root mean square. | | | |

**Supplementary Table 1:** COP movement parameters and their mathematical formulas
