# Supplementary material for: Using the center of pressure movement analysis in evaluating spontaneous movements in infants: a comparative study with general movements assessment
Source: Ital J Pediatr. 2023 Dec 20;49:165. doi: 10.1186/s13052-023-01568-8 (PMC10731817; doi:10.1186/s13052-023-01568-8)
Supplement: Supplementary file 4 — Additional file 4. Group comparisons for COP movement parameters. Shows group comparisons of COP movement parameters in Normal FMs and Absent FMs groups. [file 13052_2023_1568_MOESM4_ESM.docx]

**Supplementary Table 2:** Group comparisons for COP movement parameters

|  | | **Normal FMs** | | **Absent FMs** | |  | **Global GMA** | | **MOS-R** | |
| --- | --- | --- | --- | --- | --- | --- | --- | --- | --- | --- |
|  |  | **Mean±SD** | **Median(%25/75 IQR)** | **Mean±SD** | **Median(%25/75 IQR)** | **p^+^** | **r** | **p^#^** | **r** | **p^α^** |
| **Instantaneous velocity R** | **Std** | 228.29±110.78 | 207.79( 159.34 / 286.74) | 159.95±59.83 | 156.67( 131.03 / 215.53) | **0.035** | -0.213 | **0.032** | 0.240 | **0.015** |
|  | **RMS** | 306.71±148.20 | 272.92( 209.36 / 383.54) | 231.37±87.23 | 232.69( 183.67 / 288.81) | 0.109 | -0.176 | 0.078 | 0.240 | **0.016** |
|  | **Skewness** | 11.94±4.05 | 11.72( 9.22 / 14.14) | 14.87±6.86 | 11.57( 10.08 / 15.38) | 0.235 | 0.216 | **0.030** | -0.215 | **0.031** |
| **Instantaneous velocity X** | **Std** | 118.95±48.23 | 110.53( 86.95 / 149.67) | 110.61±44.51 | 92.14( 87.43 / 149.13) | 0.634 | -0.059 | 0.558 | 0.191 | 0.055 |
|  | **RMS** | 118.94±48.23 | 110.53( 86.93 / 149.65) | 110.60±44.51 | 92.14( 87.43 / 149.13) | 0.634 | -0.059 | 0.558 | 0.191 | 0.055 |
|  | **Skewness** | -0.23±1.26 | -0.11( -0.71 / 0.32) | 0.12±1.09 | -0.07( -0.8 / 0.5) | 0.536 | 0.096 | 0.338 | -0.060 | 0.552 |
| **Instantaneous velocity Y** | **Std** | 281.33±142.95 | 254.77( 178.27 / 350.56) | 202.33±77.63 | 213.67( 161.29 / 247.39) | 0.065 | -0.192 | 0.055 | 0.242 | **0.015** |
|  | **RMS** | 281.31±142.94 | 254.76( 178.26 / 350.54) | 202.32±77.62 | 213.67( 161.29 / 247.35) | 0.065 | -0.192 | 0.055 | 0.241 | **0.015** |
|  | **Skewness** | -3.09±2.01 | -2.92( -4.16 / -1.73) | -3.32±2.46 | -2.76( -3.58 / -1.77) | 0.895 | -0.037 | 0.711 | 0.015 | 0.879 |
| **Average velocity** | **X** | 79.36±34.71 | 73.19( 55 / 100.01) | 73.80±28.86 | 65.7( 55.27 / 99.92) | 0.816 | -0.055 | 0.584 | 0.190 | 0.057 |
|  | **Y** | 168.16±95.80 | 144.88( 102.26 / 215.37) | 133.56±54.32 | 134.03( 98.67 / 160.59) | 0.346 | -0.126 | 0.208 | 0.220 | **0.027** |
|  | **R** | 201.53±105.14 | 172.12( 128.89 / 261.28) | 166.61±65.10 | 172.71( 123.61 / 204.23) | 0.417 | -0.116 | 0.248 | 0.225 | **0.023** |
| **Velocity range** | **X** | 2115.43±894.95 | 2085.19( 1490.98 / 2600.9) | 2080.44±878.59 | 1805.3( 1338.29 / 2886.27) | 0.984 | -0.013 | 0.895 | 0.082 | 0.416 |
|  | **Y** | 6333.63±3096.02 | 5671.97( 4186.23 / 7411.71) | 4806.43±1720.54 | 4760.9( 3979.73 / 5896.88) | 0.096 | -0.172 | 0.086 | 0.198 | **0.047** |
|  | **R** | 3798.70±1987.61 | 3392.15( 2543.73 / 4442.34) | 2911.41±949.37 | 2693.91( 2567.67 / 3475.86) | 0.098 | -0.157 | 0.118 | 0.203 | **0.042** |
| **Instantaneous acceleration R** | **Std** | 8477.21±4741.53 | 7256.03( 5039.46 / 10947.99) | 6014.08±2289.51 | 5530.97( 4618.47 / 8083.82) | 0.098 | -0.181 | 0.069 | 0.190 | 0.057 |
|  | **RMS** | 8476.71±4741.25 | 7255.6( 5039.16 / 10947.34) | 6013.73±2289.38 | 5530.64( 4618.2 / 8083.33) | 0.098 | -0.181 | 0.069 | 0.190 | 0.057 |
|  | **Skewness** | -1.27±1.78 | -1.03( -2.37 / -0.35) | -0.47±1.51 | -0.57( -1.34 / 0.64) | 0.111 | 0.152 | 0.129 | -0.104 | 0.302 |
| **Instantaneous acceleration X** | **Std** | 3384.08±1547.84 | 2953.14( 2277.79 / 4343.14) | 3574.86±1558.75 | 3057.33( 2556.92 / 4775.57) | 0.556 | 0.042 | 0.679 | 0.060 | 0.551 |
|  | **RMS** | 3383.88±1547.75 | 2952.96( 2277.65 / 4342.9) | 3574.66±1558.66 | 3057.16( 2556.78 / 4775.29) | 0.556 | 0.042 | 0.679 | 0.060 | 0.551 |
|  | **Skewness** | 0.63±2.14 | 0.26( -0.56 / 2) | 1.34±3.25 | 0.73( -0.36 / 3.96) | 0.265 | 0.104 | 0.302 | -0.120 | 0.231 |
| **Instantaneous acceleration Y** | **Std** | 10777.93±6176.71 | 9281.41( 6344.5 / 13879) | 7557.98±2865.82 | 6944.35( 5883.32 / 9610.98) | 0.090 | -0.182 | 0.068 | 0.188 | 0.059 |
|  | **RMS** | 10777.29±6176.35 | 9280.87( 6344.14 / 13878.18) | 7557.53±2865.65 | 6943.94( 5882.97 / 9610.38) | 0.090 | -0.182 | 0.068 | 0.188 | 0.059 |
|  | **Skewness** | 3.76±2.86 | 3.47( 2.19 / 5.35) | 3.44±1.85 | 3.68( 2.84 / 4.14) | 0.911 | -0.039 | 0.695 | 0.030 | 0.766 |
| **Total distance (Sway Path Length)** | **X** | 13488.87±5900.09 | 12441.44( 9347.45 / 16999.69) | 12545.29±4904.87 | 11168.21( 9392.09 / 16981.96) | 0.816 | -0.055 | 0.585 | 0.190 | 0.057 |
|  | **Y** | 28583.81±16283.55 | 24627.79( 17381.07 / 36610.19) | 22703.15±9233.54 | 22780.69( 16773 / 27294.72) | 0.346 | -0.126 | 0.208 | 0.220 | **0.027** |
|  | **R** | 34255.84±17871.29 | 29257.43( 21908.4 / 44414.43) | 28320.74±11066.09 | 29357.02( 21012.37 / 34716.1) | 0.417 | -0.116 | 0.248 | 0.225 | **0.023** |
| **Instantaneous distance** | **RMS (X)** | 2.38±.96 | 2.22( 1.74 / 2.99) | 2.21±0.89 | 1.87( 1.75 / 2.98) | 0.655 | -0.059 | 0.555 | 0.191 | 0.056 |
|  | **RMS (Y)** | 5.63±2.86 | 5.1( 3.57 / 7) | 4.04±1.55 | 4.28( 3.22 / 4.95) | 0.065 | -0.192 | 0.054 | 0.240 | **0.015** |
|  | **RMS (R)** | 6.13±2.96 | 5.46( 4.19 / 7.67) | 4.63±1.74 | 4.64( 3.68 / 5.79) | 0.111 | -0.177 | 0.077 | 0.237 | **0.017** |
| **Ellipse** | **95% CI area** | 4598.85±3028.25 | 4054.02( 2346.11 / 6282.93) | 3441.69±2647.60 | 3162.2( 1642.13 / 4307.61) | 0.171 | -0.130 | 0.195 | 0.346 | **0.000** |
|  | **Minor axis** | 49.34±19.97 | 47.52( 34.81 / 63.37) | 45.04±20.60 | 46.35( 33.5 / 55.81) | 0.619 | -0.072 | 0.472 | 0.261 | **0.008** |
|  | **Major axis** | 68.83±29.23 | 65.5( 45.62 / 87.43) | 64.58±21.32 | 62.7( 51.48 / 80.46) | 0.776 | -0.051 | 0.615 | 0.243 | **0.015** |
| **Range** | **X** | 194.41±87.76 | 168.17( 136.04 / 244.62) | 177.06±66.97 | 186.74( 125.71 / 223.34) | 0.648 | -0.068 | 0.496 | 0.153 | 0.127 |
|  | **Y** | 256.20±112.30 | 235.99( 191.67 / 293.6) | 210.27±75.02 | 216.66( 181.2 / 270.63) | 0.248 | -0.142 | 0.157 | 0.284 | **0.004** |
| **Average distance** | **X** | 17.24±7.59 | 15.95( 12.38 / 20.87) | 17.92±8.46 | 17.33( 14.85 / 21.53) | 0.708 | 0.030 | 0.765 | 0.177 | 0.076 |
|  | **Y** | 19.13±9.29 | 17.68( 11.62 / 23.99) | 16.38±6.84 | 15.73( 11.34 / 20.28) | 0.394 | -0.102 | 0.308 | 0.322 | **0.001** |
|  | **R** | 28.75±11.79 | 28.05( 19.64 / 34.21) | 27.20±10.15 | 25.38( 21.6 / 34.13) | 0.692 | -0.045 | 0.655 | 0.291 | **0.003** |
| **Approximate entropy** | **X** | 1.37±0.79 | 1.27( 0.85 / 1.73) | 2.21±1.41 | 2.08( 1.36 / 2.58) | **0.044** | 0.304 | **0.002** | -0.240 | **0.016** |
|  | **Y** | 3.14±1.16 | 3.03( 2.22 / 3.94) | 4.85±2.11 | 4.27( 3.94 / 5.11) | **0.001** | 0.405 | **<0.001** | -0.332 | **0.001** |
|  | **R** | 3.07±1.19 | 3.01( 2.06 / 3.92) | 4.78±2.13 | 4.35( 3.45 / 5.04) | **0.002** | 0.397 | **<0.001** | -0.333 | **0.001** |

p^+^: Mann-Whitney U test, p^#^: Point biserial correlation test, p^α^: Spearman correlation test, IQR: Interquartile range, SD: Standard Deviation, Std: Standard Deviation,

RMS: Root mean square, MOS: Motor Optimality Score.
